# Fuzzy Counter Propagation Neural Network Control for a Class of Nonlinear Dynamical Systems

**DOI:** 10.1155/2015/719620

**Published:** 2015-08-20

**Authors:** Vandana Sakhre, Sanjeev Jain, Vilas S. Sapkal, Dev P. Agarwal

**Affiliations:** ^1^Madhav Institute of Technology & Science, Gwalior 474005, India; ^2^SGB Amravati University, Amravati 444062, India; ^3^IIT, Delhi 110001, India

## Abstract

Fuzzy Counter Propagation Neural Network (FCPN) controller design is developed, for a class of nonlinear dynamical systems. In this process, the weight connecting between the instar and outstar, that is, input-hidden and hidden-output layer, respectively, is adjusted by using Fuzzy Competitive Learning (FCL). FCL paradigm adopts the principle of learning, which is used to calculate Best Matched Node (BMN) which is proposed. This strategy offers a robust control of nonlinear dynamical systems. FCPN is compared with the existing network like Dynamic Network (DN) and Back Propagation Network (BPN) on the basis of Mean Absolute Error (MAE), Mean Square Error (MSE), Best Fit Rate (BFR), and so forth. It envisages that the proposed FCPN gives better results than DN and BPN. The effectiveness of the proposed FCPN algorithms is demonstrated through simulations of four nonlinear dynamical systems and multiple input and single output (MISO) and a single input and single output (SISO) gas furnace Box-Jenkins time series data.

## 1. Introduction

With the rapid advance of computers, the digitization has swept in all fields of science and technology with special emphasize on modeling and identification. Most of the physical processes are continuous in nature but they are generally modeled using discrete time with the use of derivative and integral as they are easily realizable. Taking these advantages, an enormous research has been focused on discrete time methods and many techniques based on linear and nonlinear discrete systems have been developed [1, 2]. Neural network has been widely used for nonlinear dynamical systems [3–5]. Various control systems like back stepping control [6, 7], sliding mode control [8, 9], and control using soft computing [10] are continuous source of interest. The nonlinear dynamical system is a generic problem which finds its application in every field of engineering. The technique based on soft computing, fuzzy logic, and neural network has also found its application in modeling of systems in various application domains. Feedforward Neural Network (FNN) is one of the most commonly used networks for this purpose; FNN with Back Propagation algorithm is another powerful network. Neural network is most popular approach due to its capability of modeling most of the nonlinear functions approximately to any arbitrary degree of accuracy [11]. Most of the systems are designed using feedforward network with gradient descent learning but the gradient descent method encounters the problem of slow convergence. This problem of convergence can be overcome by use of some Cauchy-Newton [12] Quasi-Newtonian method and Levenberg-Marquardt algorithms [13].

Optimal controllers for hybrid dynamical systems (HDS) developed using Hamilton-Jacobi-Bellman (HJB) solution method [14, 15]. Novel Lyapunov-Krasovskii functional (LKF) with triple integral time constructed for exponential synchronization of complex dynamical networks will control packet loss and additive time varying delay [16]. Control of discrete time varying system using dynamical model is difficult. To overcome this condition, new neural network approximation structure was developed to solve optimal tracking problem of nonlinear discrete time varying time system using reinforcement learning (RL) method [17]. To enhance the performance of nonlinear multivariable system, fuzzy optimal controller based Takagi-Sugeno model was developed which was used to minimize a quadratic performance index [18]. An ultimate stable training algorithm inspired by adaptive observer for black box identification of nonlinear discrete systems developed using state space recurrent neural network. The algorithm developed was using only input and output measurement in discrete time [19]. Indirect adaptive controller that uses neural network was presented for the identification and control of experimental pilot distillation column. This system is multivariable with unknown dynamics and the neural network was trained using Levenberg-Marquardt algorithm [20]. Adaptive finite time stabilization of a class of switched nonlinear systems was investigated with unknown nonlinear terms using neural network. The finite law and adaptive law were constructed using radial basis function neural network (RBFNN) to approximate the unknown packaged function. Stability analysis of RBFNN was observed to evaluate the states of the closed loop systems [21]. An adaptive fuzzy output tracking control approach was proposed in [22] for single input single output (SISO) system which is uncertain and nonlinear under arbitrary switching. An iterative learning control scheme was proposed for a class of nonlinear dynamic systems which includes holonomic systems as its subsets with linear feedback mechanism and feedforward learning strategies [23].

In this proposed work we have used instar-outstar structure based CPN with Fuzzy Competitive Learning (FCL). We have designed Fuzzy Counter Propagation Network design to control some nonlinear dynamical systems. In the FCPN, CPN model is trained by FCL algorithms. The FCL learning is used for adjusting weights and update of Best Matched Node (BMN) in discrete time nonlinear dynamical system.

The main contributions of this research are as follows:

1. This paper contributes the approximation for a class of nonlinear dynamical systems using Fuzzy Competitive Learning Based Counter Propagation Network (FCPN).
2. FCPN is employed to optimize the Mean Absolute Error, Mean Square Error, Best Fit Rate, and so forth.
3. Performance criteria of proposed FCPN for nonlinear dynamical systems are effectively improved by compensating reference signal and controller signal.

The paper is organized as follows. In Section 2, problem formulations for a class of nonlinear dynamical systems are given.  Section 3 contains description of Feedforward Neural Network (FNN) with Back Propagation Network, Dynamic Network, and Fuzzy Learning Based Counter Propagation Network. Dynamical learning for CPN and optimal learning of dynamical system are presented in Section 4. The simulation results and comparison of different nonlinear dynamical models are presented in Section 5.  Section 6 gives conclusion of the study.

## 2. Problem Formulation

Let us consider four models of discrete time nonlinear dynamical systems [24] for single input single output (SISO) and multiple input and single output (MISO) system considered in this paper and they are described by difference equations (1)–(4) and Box-Jenkins time series data [10]. Let *f* : *R* ^*n*^ → *R* and *g* : *R* ^*m*^ → *R* be the nonlinear continuous differentiable function of Model I–Model IV approximated by FCPN to the desired degree of accuracy. Once the system has been parameterized, the performance evaluation has been carried out by FCPN for (1) to (4).

Model I:

(1) yk+1=∑i=0n−1αiyk−i+guk,…,uk−m+1.

Model II:

(2) yk+1=fyk,…,yk−n+1+∑i=0m−1βiuk−i.

Model III:

(3) yk+1=fyk,…,yk−n+1+guk,…,uk−m+1.

Model IV:

(4) yk+1=fyk,…,yk−n+1;  uk,…,uk−m+1,

where [*u*(*k*), *y*(*k*)] represent the input-output pair of the system at time *k* and their order is represented by (*n*, *m*). FCL is used to learn the system defined for (1) to (4) and the performance of the FCPN can be measured by error function given in

(5) $$\begin{array}{c} E\left(\;k\;\right)=\frac{1}{2}{\left[\;y\left(\;k\;\right)-\overset{-}{y}\left(\;k\;\right)\;\right]}^{2}, \end{array}$$

where *u*(*k*) is the input, *y*(*k*) is the system output, $\overset{-}{y}(k)$ is neural network output, and (5) can be written for neural controller as

(6) $$\begin{array}{c} E^{c}=\frac{1}{P}\overset{P}{\underset{p=1}{\sum }}{\left[\;y\left(\;k\;\right)-{\overset{-}{y}}^{c}\left(\;k\;\right)\;\right]}^{2}, \end{array}$$

where ${\overset{-}{y}}^{c}(k)$ is neural controller output and (6) is known as minimized error function and *P* is total number of input patterns.

## 3. Back Propagation Network (BPN)

**Figure 1:**
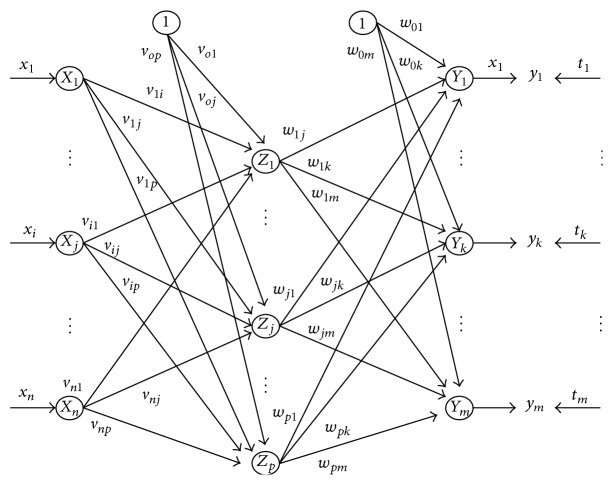
General architecture of BPN.

A neural network is one of the most popular intelligent systems which has capability to approximate calculated and target value at an arbitrary degree of accuracy. BPN is one of the most commonly used networks for this purpose. The BPN consists of input layer, hidden layer, and output layer and possesses weighted interconnections. The BPN is based on supervised learning. This method is used where error is propagated back to the hidden layer. The aim of this neural network is to train the net to achieve a balance between the net's ability to respond (memorization) and its ability to give reasonable response to the input that is similar but not identical to the one that is used in training (generalization). For a given set of training input-output pairs, this network provides a procedure for changing weights to classify the given input patterns correctly. The weight update algorithm is based on gradient descent method [25]. The network architecture is shown in Figure 1.

### 3.1. Dynamic Network

Neural network can be classified into two categories: (i) Static and (ii) Dynamic. In Static Neural Network, output can be calculated directly from input through feedforward network. But in Dynamic Network output depends on current or previous inputs, outputs, or states of network. Where the current output is function of current input and previous output, it is known as recurrent (feedback) network. Training process of Static and Dynamic Network can be differentiated by use of gradient or Jacobian which is computed. Dynamic Network contains delays and operates on a sequence of inputs. Dynamic Networks can have purely feedforward connections or they can have some recurrent connections. They can be trained using Dynamic Back Propagation Network [26].

The general equation of the net input *n* ^*m*^(*t*) for *m*th layer is given by

(7) nmt=∑l∈Lmf ∑d∈DLm·lIWm·ldalt−d+∑l∈Im ∑d∈DIm·lIWm·ldblt−d+bm,

where *b* ^*l*^(*t*) is the *l*th input vector at time *t*, IW^*m*,*l*^ is the input weight between *l*th and *m*th layer, LW^*m*,*l*^ is the layer weight between *l*th and *m*th layer, and *b* ^*m*^ is the bias vector for layer *m*. DL_*m*,*l*_ is the set of all delays in the Tapped Delay Line (TDL) between *l*th and *m*th layer and DI_*m*,*l*_ is the set of all delays in the TDL between input *l* and *m*th layer, *I*_*m*_ is the set of indices of input vectors that connect to layer *m*, and *L*_*m*_^*f*^ is the set of indices of layers that connect to layer *m*. The output of *m*th layer at time *t* is computed as

(8) $$\begin{array}{c} a^{m}\left(\;t\;\right)=f^{m}\left(\;n^{m}\left(\;t\;\right)\;\right). \end{array}$$

Network has a TDL on the input with DI_1,1_ = {0, 1, 2} and its output is represented as

(9) at=fnt=∑dIWd∗pt−d,at=fiw1,10pt+iw1,11pt−1+iw1,12pt−2.

**Figure 2:**
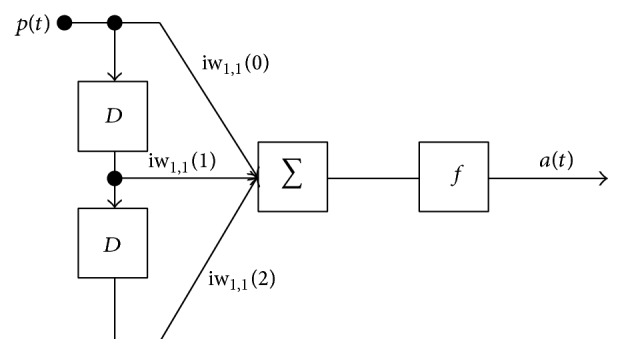
Simple architecture of Dynamic Adaptive Linear Neuron.

One simple architecture with delay DI_1,1_ = {0, 1, 2} for feedforward Dynamic Network is shown in Figure 2 known as Dynamic Adaptive Linear Neuron.

### 3.2. Counter Propagation Network (CPN)

It is multilayer feedforward network based on the combination of the input, competitive, and output layers. Model of CPN is instar-outstar. It is three-layer neural network that performs input-output data mapping, that is, producing output in the response to an input vector on the basis of Fuzzy Competitive Learning. In CPN the connection between input layer and competitive layer is instar structure and connection between competitive and output layer is outstar structure. Counter Propagation Network involves two-stage training process. In first stage input vector is clustered on the basis of Euclidean distance and the performance of network is improved using linear topology. Using Euclidean distance between input and weight vector, we evaluated BMN. In phase II, the desired response is obtained by adapting the weights from competitive layer to output layer [27]. Let $x=\left[\begin{array}{cccc} x_{1} & x_{2} & \cdots & x_{n} \end{array}\right]$ and $y=\left[\begin{array}{cccc} y_{1}^{*} & y_{2}^{*} & \cdots & y_{m}^{*} \end{array}\right]$ be input and desired vector, respectively, let *v*_*ij*_ be weight of input layer and competitive layer, where 1 ≤ *i* ≤ *n* and 1 ≤ *j* ≤ *p*, and let *w*_*jk*_ be the weight between competitive layer and output layer, where 1 ≤ *k* ≤ *m*. Euclidean distance between input vector *x* and weight vector *v*_*ij*_ is

(10) $$\begin{array}{c} D_{j}=\overset{n}{\underset{i=1}{\sum }}{\left(\;x_{i}-v_{ij}\;\right)}^{2},\;\text{where }j=1,2,\ldots ,p. \end{array}$$

**Figure 3:**
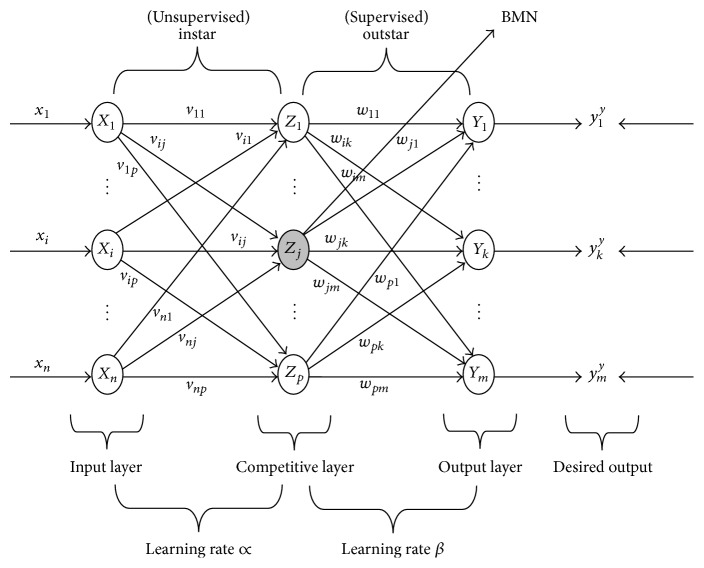
Architecture of Counter Propagation Network.

The architecture of CPN is shown in Figure 3.

### 3.3. Fuzzy Competitive Learning (FCL)

FCL is used for adjusting instar-outstar weights and update of BMN for discrete time nonlinear dynamical systems. Learning of CPN is divided into two phases: Fuzzy Competitive Learning phase (unsupervised) and Grossberg Learning phase (supervised) [28–31]. CPN is efficient network for mapping the input vector of size *n* placed in clusters of size *m*. Like traditional self-organizing map, the weight vector for every node is considered as a sample vector to the input pattern. The process is to determine which weight vector (say *J*) is more similar to the input pattern chosen as BMN. First phase is based on closeness between weight vector and input vector and second phase is weight update between competitive and output layer for desired response.

Let *v*_*ij*_ be weight of input node *i* to neuron *j* and let *w*_*jk*_ be the weight between competitive node *j* and neuron *k*. We propose a new learning rate calculation method for use of weight update:

(11) $$\begin{array}{c} \propto \left(\;J\;\right)=\frac{\sum_{i=1}^{n}{\left(v_{iJ}-x_{i}\right)}^{2}}{\sum_{j=1}^{m}\sum_{i=1}^{n}{\left(v_{iJ}-x_{i}\right)}^{2}}, \end{array}$$

where *J* denotes the index of BMN and ∝ is known as Fuzzy Competitive Learning rate.

Fuzzy Competitive Learning phase is described as follows.

#### 3.3.1. Training Algorithm of FCL

*Phase I (for determination of BMN)*. There are steps for training of CPN as follows.

**Step 1 .**

Initialize instar-outstar weights.

**Step 2 .**

Perform Steps 3–8 until stopping criteria for Phase I training fail. The stopping condition may be fixed number of epochs or learning rate has reduced to negligible value.

**Step 3 .**

Perform Steps 4–6 for all input training vector *X*.

**Step 4 .**

Set the *X*-input layer activation to vector *X*.

**Step 5 .**

Compute the Best Matched Node (BMN) (*J*) using Euclidean distance; find the node *Z*_*J*_ whose distance from the input pattern is minimum. Euclidean distance is calculated as follows:

(12) $$\begin{array}{c} D_{j}=\overset{n}{\underset{i=1}{\sum }}{\left(\;x_{i}-v_{ij}\;\right)}^{2},\;\text{where }j=1,2,\ldots ,p. \end{array}$$

Minimum net input:

(13) $$\begin{array}{c} z_{inJ}=\overset{n}{\underset{i=1}{\sum }}x_{i}v_{iJ}. \end{array}$$

**Step 6 .**

Calculate Fuzzy Competitive Learning (14) rate for weight update using BMN.

**Step 7 .**

We update weight for unit *Z*_*J*_:

(14) $$\begin{array}{c} v_{iJ}\left(\;new\;\right)=v_{iJ}\left(\;old\;\right)+\propto h\left(\;J;i,j\;\right)\left(\;x_{i}-v_{iJ}\;\right), \end{array}$$

where *i* = 1,2, 3,…, *n* and *h*(·; ·) is neighborhood function around BMN. Consider

(15) hJ;i,j=exp⁡−vij−viJ2σ−2t,where  hJ;i,j∈0,1,

(16) σ−t=σ0exp⁡−tT.

**Step 8 .**

Test the stopping criteria.

*Phase II (to obtain desired response)*

**Step 1 .**

Set activation function to (*x*, *y*) input and output layer, respectively.

**Step 2 .**

Update the BMN (*J*) (Step 5 from Phase I). Also update the weights into unit *Z*_*J*_ as

(17) $$\begin{array}{c} v_{iJ}\left(\;new\;\right)=v_{iJ}\left(\;old\;\right)+\alpha h\left(\;J;i,j\;\right)\left[\;x_{i}-v_{iJ}\left(\;old\;\right)\;\right]. \end{array}$$

**Step 3 .**

Update the weights from node *Z*_*J*_ to the output unit as

(18) wJknew=wJkold+βhJ;j,kyj−wJkoldfor  1≤k≤m.

**Step 4 .**

Learning rate *β* is calculated as

(19) $$\begin{array}{c} \beta \left(\;J\;\right)=\frac{\sum_{k=1}^{m}{\left({y_{k}-w}_{Jk}\right)}^{2}}{\sum_{j=1}^{p}\sum_{k=1}^{m}{\left({y_{k}-w}_{jk}\right)}^{2}}. \end{array}$$

**Step 5 .**

Decrease the rate of learning s.t.  *β* = *β* − *ε*, where *ε* is small positive number.

**Step 6 .**

Test the stopping condition for Phase II (i.e., fixed number of epochs or its learning rate has reduced to negligible value).

#### 3.3.2. Testing Phase (to Test FCPN)

**Step 1 .**

Set the initial weights, that is, the weights obtained during training.

**Step 2 .**

Apply FCPN to the input vector *X*.

**Step 3 .**

Find unit *J* that is closest to vector *X*.

**Step 4 .**

Set activations of output units.

**Step 5 .**

Apply activation function at *y*_*k*_, where *y*_*k*_ = ∑_*j*_ *z*_*j*_ *w*_*jk*_.

## 4. Dynamical Learning for CPN

Learning stabilities are fundamental issues for CPN; however there are few studies on the learning issue of FNN. BPN for learning is not always successful because of its sensitivity to learning parameters. Optimal learning rate always changes during the training process. Dynamical learning of CPN is carried out using Lemmas 1, 2, and 3.

*Assumption*. Let us assume *ϕ*(*x*) is a sigmoid function if it is bounded continuous and increasing function. Since input to the neural network in this model is bounded, we consider Lemmas 1 and 2 given below.

**Lemma 1 .**

Let *ϕ*(*x*) be a sigmoid function and let *Ω* be a compact set in *ℝ* ^*n*^, and *f* : *ℝ* ^*n*^ → *ℝ* on *Ω* is a continuous function and, for arbitrary ∈>0, ∃ integer *N* and real constants *c*_*i*_, *θ*_*i*_, and *w*_*ij*_, *i* = 1, 2, …, *N*, *j* = 1, 2, …, *n*, such that

(20) $$\begin{array}{c} \overset{-}{f}\left(\;x_{1},x_{2},\ldots ,x_{n}\;\right)=\overset{N}{\underset{i=1}{\sum }}c_{i}\emptyset \left(\;\overset{n}{\underset{j=1}{\sum }}w_{ij}x_{i}-\theta_{i}\;\right) \end{array}$$

satisfies

(21) $$\begin{array}{c} \underset{x\in \Omega }{\max}\left\|\;f\left(\;x_{1},x_{2},\ldots ,x_{n}\;\right)-\overset{-}{f}\left(\;x_{1},x_{2},\ldots ,x_{n}\;\right)\;\right\|<\in . \end{array}$$

Using Lemma 1, dynamical learning for a three-layer CPN can be formulated where the hidden layer transfer functions are *ϕ*(*x*) and transfer function at output layer is linear.

Let all the vectors be column vectors and superscript *d*_*p*_^*k*^ refers to specific output vectors component.

Let *X* = [*x*_1_, *x*_2_,…, *x*_*p*_] ∈ *ℝ* ^*L*×*P*^, *Y* = [*y*_1_, *y*_2_,…, *y*_*p*_] ∈ *ℝ* ^*H*×*P*^, *O* = [*o*_1_, *o*_2_,…, *o*_*p*_] ∈ *ℝ* ^*K*×*P*^, *D* = [*d*_1_, *d*_2_,…, *d*_*p*_] ∈ *ℝ* ^*K*×*P*^ be the input, hidden, output, and desired vector, respectively, where *L*, *H*, and *K* denote the number of input, hidden, and output layer neurons. Let *V* and *W* represent the input-hidden and hidden-output layer weight matrix, respectively. The objectives of the network training minimize error function *J*, where *J* is

(22) $$\begin{array}{c} J=\frac{1}{2PK}\overset{P}{\underset{p=1}{\sum }}\;\overset{K}{\underset{k=1}{\sum }}{\left(\;o_{p}^{k}-d_{p}^{k}\;\right)}^{2}. \end{array}$$

### 4.1. Optimal Learning of Dynamical System

Consider a three-layer CPN; the network error matrix is defined by the error between differences of desired and FCPN output at any iteration and given as

(23) $$\begin{array}{c} E_{t}=O_{t}-D=W_{t}Y_{t}-D=W_{t}Y_{t}X-D. \end{array}$$

The objective of the network for minimization of error given in (24) is defined as follows:

(24) s.t. J=12PKTrEtEtT,

where *T*_*r*_ represent the trace of matrix. Using gradient descent method updated weight is given by

(25) Wt+1=Wt−βt∂J∂Wt,Vt+1=Vt−βt∂J∂Vt,

or

(26) Wt+1=Wt−βtPKEtYtT,Vt+1=Vt−βtPKWtTEtXT.

Using (23)–(26), we have

(27) $$\begin{array}{c} E_{t+1}E_{t+1}^{T}=\left(\;W_{t+1}Y_{t+1}-D\;\right){\left(\;W_{t+1}Y_{t+1}-D\;\right)}^{T}. \end{array}$$

To obtain minimum error for multi-layer FNN in above equation (28) after simplification, we have

(28) Jt+1−Jt=12PKgβ,J=12PK∑p=1P ∑k=1Kopk−dpk2,Et+1Et+1T=Wt+1Vt+1X−DWt+1Vt+1X−DT=Wt−βtPKEtYtTVt−βtPKWtTEtXTX−DWt−βtPKEtYtTVt−βtPKWtTEtXTX−DT=Et−βtPKWtWtTEtXTX+EtYtTVtX+βt2PK2EtYtTWtTEtXTX∗Et−βtPKWtWtTEtXTX+EtYtTVtX+βt2PK2EtYtTWtTEtXTXT=EtEtT−βtPKEtWtWtTEtXTXT+EtEtYtTVtXT+WtWtTEtXTXEtT+EtYtTVtXEtT+βt2PK2EtEtYtTWtTEtXTXT+EtYtTWtTEtXTXEtT+EtYtTVtXWtWtTEtXTXT+WtWtTEtXTXEtYtTVtXT+EtYtTVtXEtYtTVtXT+WtWtTEtXTXWtWtTEtXTXT−βt3PK3WtWtTEtXTXEtYtTWtTEtXTX+EtYtTVtXEtYtTWtTEtXTXT+EtYtTWtTEtXTXWtWfTEtXTXT+EtYtTWtTEtXTXEtYtTVtXT+βt4PK4EtYtTWtTEtXTXEtYtTWtTEtXTXT.

For simplification, omit subscript *t*, we have; that is,

(29) $$\begin{array}{c} J_{t+1}-J_{t}=\frac{1}{2PK}\left(\;A\beta^{4}+B\beta^{4}+C\beta^{4}+M\beta \;\right) \end{array}$$

where

(30) A=1PK4TrEYTWEXTXEYTWTEXTXT,B=1PK3TrWWTEXTXEYTWTEXTXT+EYTVXEYTWTEXTXT+EYTWTEXTXWWTEXTXT+EYTWTEXTXEYTVX,C=1PK2TrEYTWTEXTXT+EYTWEXTXET+EYTVXWWTEXTXT+WWTEXTX+EYTVXT+EYTVXEYTVXT+WWTEXTXWWTEXTXT,M=1PKTrEWWTEXTXT+EEYTVXT+WWTEXTX+EYTVXET,

where

(31) gβ=Aβ4+Bβ3+Cβ2+Mβ,

(32) ∂g∂β=4Aβ3+aβ2+bβ+c,

where *a* = 3*B*/4*A*, *b* = 2*C*/4*A*, and *c* = *M*/4*A*.

**Lemma 2 .**

For solution of general real cubic equation, one uses the following lemma:

(33) fx=x3+ax2+bx+c,Let  D=−27c2+18cab+a2b2−4a3b3−4b3,

where *D* is discriminant of *f*(*x*).

Then we have the following:

(1) If *D* < 0, *f*(*x*) has one real root.
(2) If *D* ≥ 0, *f*(*x*) has three real roots:
  (a) *D* > 0; *f*(*x*) has three different real roots,
  (b) *D* = 0, 6*b* − 2*a* ^2^#0; *f*(*x*) has one single root and one multiple root,
  (c) *D* = 0, 66 − 2*a* ^2^ = 0; *f*(*x*) has one root of three multiplicities.

**Lemma 3 .**

For given polynomial *g*(*β*) given (31) if optimum *β* = {*β*_*i*_∣*g*(*β*_*i*_) = min⁡(*g*(*β*_1_), *g*(*β*_2_), *g*(*β*_3_)), *i* ∈ {1, 2, 3}}, where *β*_*i*_ is real root of  ∂*g*/∂*β*, the optimum (*β*) is the optimal learning rate and this learning process is stable.

**Proof**

To find stable learning range of *β*, consider that Lyapunov function is

(34) Vt=Jt2,ΔVt=Jt+12−Jt2if  ΔVt<0,

and then dynamical system is guaranteed to be stable if Δ*V*_*t*_ < 0; that is, *J*_*t*+1_ − *J*_*t*_ = (1/2*PK*)(*Aβ* ^4^ + *Bβ* ^4^ + *Cβ* ^4^ + *Mβ*) < 0 (29), where *β* is learning rate, since in the training process input matrix remains the same during the whole training process. To find the range of *β*, which satisfy *g*(*β*) = (*Aβ* ^4^ + *Bβ* ^3^ + *Cβ* ^2^ + *Mβ*) < 0. Since ∂*g*/∂*β*, where has at least one real root (Lemma 2) and one of them must give optimum *g*(*β*). Obviously minimum value of *g*(*β*) gives the largest reduction in *J*_*t*_ at each step of learning process. Equation (31) shows that *g*(*β*) has two or four real roots, one including *β* = 0 (Lemma 2), such that minimum value of *β* shows largest reduction in error at two successive times and minimum value is obtained by differentiating (31) with respect to *β* we have from (32):

(35) $$\begin{array}{c} \frac{\partial g}{\partial \beta }=4A\left(\;\beta^{3}+a\beta^{2}+b\beta +C\;\right), \end{array}$$

where *a* = 3*B*/4*A*, *b* = 2*C*/4*A*, and *c* = *M*/4*A*.

Solving ∂*g*/∂*β* = 0 gives *β* which minimizes error in (5).

## 5. Simulation Results

To demonstrate the effectiveness and merit of proposed FCPN, simulation results are presented and discussed. Four different nonlinear dynamical systems and one general benchmark problem known as Box-Jenkins model with time series data are considered.

### 5.1. Performance Criteria

For accessing the performance criteria of FCPN and comparing with Dynamic and Back Propagation Network, we have evaluated various errors as given below. In case of four dynamical models, it is recommended to access criterion such as subset of the following.

Given *N* pair of data points $\left(y\left(k\right),\overset{-}{y}\left(k\right)\right)$, where *y*(*k*) is output of system and $\overset{-}{y}\left(k\right)$ is output of controller, the Maximum Absolute Error (MAE) is

(36) $$\begin{array}{c} J_{MAE}=J_{max}=\underset{1\leq k\leq N}{\max}\left|\;y\left(\;k\;\right)-\overset{-}{y}\left(\;k\;\right)\;\right|, \end{array}$$

the Sum of Squared Errors (SSE) is

(37) $$\begin{array}{c} J_{SSE}=\overset{N}{\underset{k=1}{\sum }}{\left(\;y\left(\;k\;\right)-\overset{-}{y}\left(\;k\;\right)\;\right)}^{2}, \end{array}$$

the Mean Squared Error (MSE) is

(38) $$\begin{array}{c} J_{MSE}=\frac{1}{N}\overset{N}{\underset{k=1}{\sum }}{\left(\;y\left(\;k\;\right)-\overset{-}{y}\left(\;k\;\right)\;\right)}^{2}, \end{array}$$

and/or the Root Mean Squared Error (RMSE) is

(39) $$\begin{array}{c} J_{RMSE}=\sqrt{J_{MSE}}. \end{array}$$

The measure Variance Accounting Factor (VAF) is

(40) $$\begin{array}{c} J_{VAF}=\left(\;1-\frac{Var\left(y\left(k\right)-\overset{-}{y}\left(k\right)\right)}{Var\left(y\left(k\right)\right)}\;\right)100\%. \end{array}$$

A related measure is the Normalized Mean Squared Error (NMSE):

(41) $$\begin{array}{c} J_{NMSE}=\frac{\sum_{k=1}^{N}{\left(y\left(k\right)-\overset{-}{y}\left(k\right)\right)}^{2}}{\sum_{k=1}^{N}{\left(y\left(k\right)-\overset{-}{y}\right)}^{2}}, \end{array}$$

and the Best Fit Rate (BFR) is

(42) $$\begin{array}{c} J_{BFR}=\left(\;1-\frac{\sqrt{\sum_{k=1}^{N}{\left(y\left(k\right)-\overset{-}{y}\left(k\right)\right)}^{2}}}{\sum_{k=1}^{N}{\left(y\left(k\right)-\hat{y}\right)}^{2}}\;\right)100\%. \end{array}$$

**Example 1 .**

The nonlinear dynamical system [24] is governed by the following difference equation:

(43) $$\begin{array}{c} y\left(\;k+1\;\right)=0.3y\left(\;k\;\right)+0.6y\left(\;k-1\;\right)+f\left[\;u\left(\;k\;\right)\;\right]. \end{array}$$

**Figure 4:**
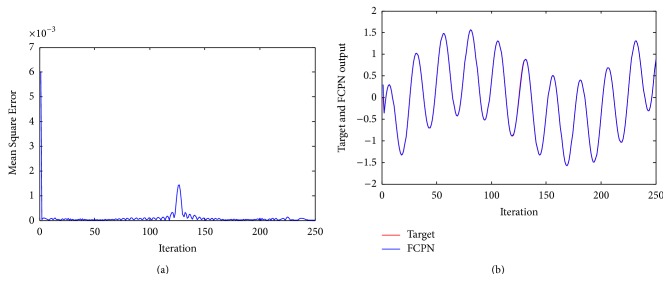
(a) Mean Square Error of system (Example 1) using FCPN. (b) Performance of the controller using FCPN algorithm for Example 1.

The nonlinear function in the system in this case is *f*(*u*) = *u* ^3^ + 0.3*u* ^2^ − 0.4*u*, where *y* and *u* are uniformly distributed in [−2, 2]. The objective of Example 1 is to control the system to track reference output given as 250 sample data points. The model has two inputs *y*(*k*) and *u*(*k*) and single output *y*(*k* + 1) and the system identification was initially performed with the system input being uniformly distributed over [−2, 2]. The FCPN controller uses two inputs *y*(*k*) and *u*(*k*) to produce output $\bar{y}\left(k+1\right)$. FCPN model governed by the difference equation $\bar{y}\left(k+1\right)=0.3y\left(k\right)+0.6y\left(k-1\right)+N[u\left(k\right)]$ was used. Figures 4(a) and 4(b) show the error $e\left(k+1\right)=y\left(k+1\right)-\bar{y}\left(k+1\right)$ and outputs of the dynamical system and the FCPN. As can be seen from the figure, the identification error is small even when the input is changed to a sum of two sinusoids *u*(*k*) = sin⁡(2*πk*/250) + sin⁡(2*πk*/25) at *k* = 250.

Table 1 includes various calculated errors of different NN models for Example 1. It can be seen from the table that various errors calculated for FCPN are minimum as compared to the DN and BPN.

**Example 2 .**

The nonlinear dynamical system [24] is governed by the following difference equation:

(44) $$\begin{array}{c} y\left(\;k+1\;\right)=f\left[\;y_{p}\left(\;k\;\right),y_{p}\left(\;k-1\;\right)\;\right]+u\left(\;k\;\right), \end{array}$$

where

(45) fypk,ypk−1=ypkypk−1ypk+2.51+yp2k+yp2k−1.

The objective of (44) is to control the system to track reference output given 100 sample data points. The model has two inputs *y*(*k*) and *u*(*k*) and single output *y*(*k* + 1) and the system identification was initially performed with the system input being uniformly distributed over [−2, 2]. Training and testing samples contain 100 sample data points. The FCPN controller uses two inputs *y*(*k*) and *u*(*k*) to output $\bar{y}\left(k+1\right)$. FCPN network discussed earlier is used to identify the system from input-output data and is described by the equation

(46) $$\begin{array}{c} \bar{y}\left(\;k+1\;\right)=N\left[\;y\left(\;k\;\right),y\left(\;k-1\;\right)\;\right]+u\left(\;k\;\right). \end{array}$$

**Figure 5:**
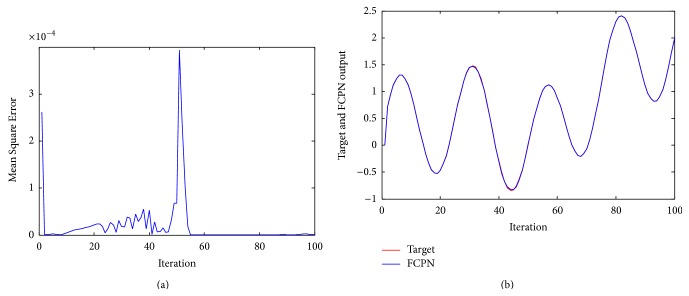
(a) Mean Square Error of system (Example 2) using FCPN. (b) Performance of the controller using FCPN algorithm for Example 2.

For FCPN, the identification process involves the adjustment of the weights of *N* using FCL. FCPN identifier needs some prior information concerning the input-output behavior of the system before identification can be undertaken. FCL is used to adjust the weights of the neural network so that the error $e\left(k+1\right)=y\left(k+1\right)-\bar{y}(k+1)$ is minimized as shown in Figure 5(a). The behavior of the FCPN model for (16) is shown in Figure 5(b). The input *u*(*k*) was assumed to be a random signal uniformly distributed in the interval [−2, 2]. The weights in the neural network were adjusted.

Table 2 includes various calculated errors of different NN models for Example 2. It can be seen from the table that various errors calculated for FCPN are minimum as compared to the DN and BPN.

**Example 3 .**

The nonlinear dynamical system [23] is governed by the following difference equation:

(47) $$\begin{array}{c} y\left(\;k+1\;\right)=\frac{y\left(k\right)}{1+y{\left(k\right)}^{2}}+u^{3}\left(\;k\;\right), \end{array}$$

which corresponds to *f*[*y*(*k*)] = *y*(*k*)/(1 + *y*(*k*)^2^) and *g*[*u*(*k*)] = *u* ^3^(*k*). FCPN network equation (47) is used to identify the system from input-output data. Weights in the neural networks were adjusted for every instant of discrete time steps.

**Figure 6:**
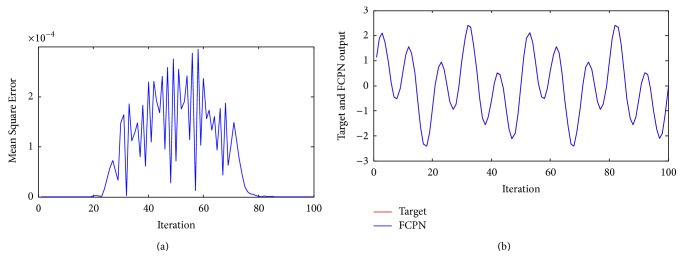
(a) Mean Square Error of system (Example 3) using FCPN. (b) Performance of the controller using FCPN algorithm for Example 3.

The objective of (47) is to control the system to track reference output given 100 sample data points. The model has two inputs *y*(*k*) and *u*(*k*) and single output *y*(*k* + 1) and the system identification was initially performed with the system input being uniformly distributed over [−2, 2]. Training and testing samples contain 250 sample data points. The FCPN controller uses two inputs *y*(*k*) and *u*(*k*) to output $\bar{y}\left(k+1\right)$. The input data is generated using uniform distribution in interval [−2, 2]. The function $\bar{f}$ obtained by FCPN is used to adjust the weights of the neural network so that the error $e\left(k+1\right)=y\left(k+1\right)-\bar{y}(k+1)$ is minimized as shown in Figure 6(a). In Figure 6(b), the desired outputs of the system as well as the FCPN model are shown and are seen to be indistinguishable.

Table 3 includes various calculated errors of different NN models for Example 3. It can be seen from the table that various errors calculated for FCPN are minimum as compared to the DN and BPN.

**Example 4 .**

The nonlinear dynamical system [24] is governed by the difference equation (48). Generalized form of this equation is Model IV. In this example, the system is assumed to be of the form

(48) ypk+1=fypk,ypk−1,ypk−2,uk,uk−1,

where the unknown function *f* has the form

(49) $$\begin{array}{c} f\left[\;x_{1},x_{2},x_{3},x_{4},x_{5}\;\right]=\frac{x_{1}x_{2}x_{3}x_{5}\left(x_{3}-1\right)+x_{4}}{1+x_{3}^{2}+x_{2}^{2}}. \end{array}$$

**Figure 7:**
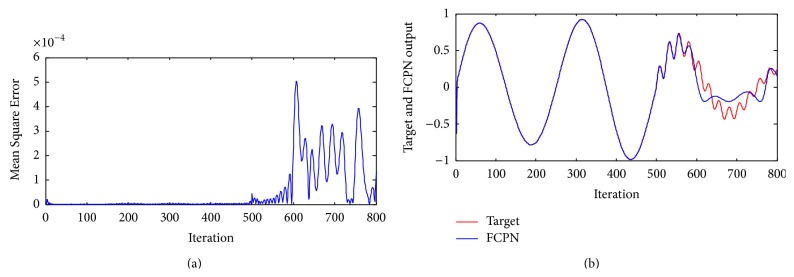
(a) Mean Square Error of system (Example 4) using FCPN. (b) Performance of the controller using FCPN algorithm for Example 4.

In the identification model, which is approximated by FCPN, Figure 7(b) shows the output of the system and the model when the identification procedure carried random input signal uniformly distributed in the interval [−1, 1]. The performance of the model is studied and the error *e*(*k* + 1) = *N*[*y*_*p*_(*k* + 1)] is minimized as shown in Figure 7(a). In Figure 7(b), the outputs of the system as well as output of FCPN model are shown. Input to the system and the identified model is given by *u*(*k*) = sin⁡(2*πk*/250) for *k* ≤ 500 and *u*(*k*) = 0.8sin⁡(2*πk*/250) + 0.2sin⁡(2*πk*/25) for *k* > 500.

Table 4 includes various calculated errors of different NN models for Example 4. It can be seen from the table that various errors calculated for FCPN are minimum as compared to the DN and BPN.

**Example 5 .**

In this example we have used Box-Jenkins time series [32], of 296 pairs of data measured from a gas furnace system with single input *u*(*t*) being gas flow rate and single output *y*(*t*) being CO_2_ concentration in outlet gas. Training samples and testing samples contained 196 and 100 data points, respectively. The FCPN uses the two inputs, the current state *y*(*k*) and the desired state *y* ^*d*^(*k*), to produce an output which is $\bar{y}(k)$ [33].

**Figure 8:**
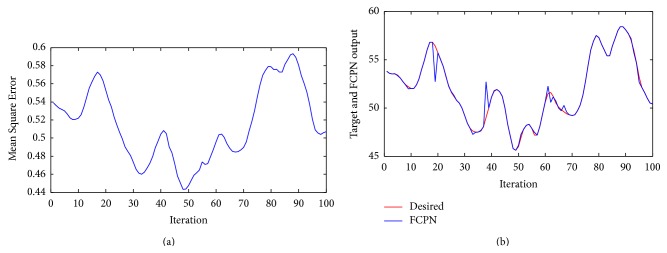
(a) Mean Square Error of system (Example 5) using FCPN. (b) Performance of the controller using FCPN algorithm for Example 5.

Figure 8(a) shows the mean squared control errors of FCPN methods.  Figure 8(b) shows the performance of the controller with FCPN algorithm. Result shows that FCPN algorithm enable us to appropriate the approximation using fuzzy learning as given in equation (11) of Box Jenkins time series data, based on calculation of BMN.

Table 5 shows BFR (%) various NN models for all Examples 1–5, respectively. It can be observed from Table 5 that the Best Fit Rate found for FCPN is maximum as compared to DN and BPN which shows better performance of the FCPN network for nonlinear system.

## 6. Conclusions

FCPN is a neural network control method which was developed and presented. It is based on the concept of combining FCL algorithm and CPN. The key ideas explored are the use of the FCL algorithm for training the weights of the instar-outstar. The performances of the FCPN controller based training are tested using four nonlinear dynamical systems and on time series (Box-Jenkins) data and compared with the Dynamic Network and standard Back Propagation algorithm. The comparative performances of the FCPN algorithm and Dynamic Network, such as the number of iterations and performance functions like MAE, MSE, SSE, NMSE, BFR, and so forth, of FCPN and Dynamic Network error are summarized in Tables 1–4. It can be seen that the FCPN algorithm gives minimum errors as compared to the Dynamic Network and standard Back Propagation Network for all four models of nonlinear dynamical systems and Box-Jenkins time series data. Results obtained from FCPN were compared for various errors and it is clearly shown that FCPN works much better for the control of nonlinear dynamical systems.

## Figures and Tables

**Table 1:** Calculated various errors for Example 1.

NN model	Different error calculation				
	MAE	SSE	MSE	RMSE	NMSE
FCPN	1.3364	0.1143	4.5709*e* − 004	0.0214	7.1627*e* − 004
Dynamic	5.8231	3.1918	0.0128	0.1130	0.0028
BPN	38.0666	48.1017	0.4810	0.6936	0.1035

**Table 2:** Calculated various errors for Example 2.

NN models	Different error calculation				
	MAE	SSE	MSE	RMSE	NMSE
FCPN	0.1956	0.0032	3.2328*e* − 005	0.0057	2.7164*e* − 005
Dynamic	9.5152	2.9856	0.0299	0.1728	0.0308
BP	10.1494	4.5163	0.0452	0.2125	0.0323

**Table 3:** Calculated various errors and BFR for Example 3.

NN model	MAE	SSE	MSE	RMSE	NMSE
FCPN	0.3519	0.0032	3.1709*e* − 005	0.0056	5.6371*e* − 006
Dynamic	5.5652	3.6158	0.0362	0.1902	0.0223
BP	42.6524	46.9937	0.4699	0.6855	0.2892

**Table 4:** Calculated various errors and BFR for Example 4.

NN models	MAE	SSE	MSE	RMSE	NMSE
FCPN	30.4348	2.1931	0.0027	0.0524	0.0186
Dynamic	32.8952	6.4059	0.0080	0.0895	0.0276
BP	53.1988	13.4174	0.0168	0.1295	0.0844

**Table 5:** BFR (%) values of NN models for various examples.

NN models	BFR (%)			
	Example 1	Example 2	Example 3	Example 4
FCPN	97.32	99.48	99.76	86.35
DN	94.69	79.32	85.08	83.39
BPN	67.83	79.28	46.2234	70.94
